# IoT-Based Healthcare-Monitoring System towards Improving Quality of Life: A Review

**DOI:** 10.3390/healthcare10101993

**Published:** 2022-10-11

**Authors:** Suliman Abdulmalek, Abdul Nasir, Waheb A. Jabbar, Mukarram A. M. Almuhaya, Anupam Kumar Bairagi, Md. Al-Masrur Khan, Seong-Hoon Kee

**Affiliations:** 1 Faculty of Electrical & Electronic Engineering Technology, Universiti Malaysia Pahang, Pekan 26600, Malaysia; 2 Faculty of Engineering and Computing, University of Science & Technology, Aden 8916162, Yemen; 3 School of Engineering and the Built Environment, Birmingham City University, Birmingham B4 7XG, UK; 4 Computer Science and Engineering Discipline, Khulna University, Khulna 9208, Bangladesh; 5 Department of ICT Integrated Ocean Smart Cities Engineering, Dong-A University, Busan 49315, Korea

**Keywords:** IoT, IoWT, healthcare, monitoring, remote

## Abstract

The Internet of Things (IoT) is essential in innovative applications such as smart cities, smart homes, education, healthcare, transportation, and defense operations. IoT applications are particularly beneficial for providing healthcare because they enable secure and real-time remote patient monitoring to improve the quality of people’s lives. This review paper explores the latest trends in healthcare-monitoring systems by implementing the role of the IoT. The work discusses the benefits of IoT-based healthcare systems with regard to their significance, and the benefits of IoT healthcare. We provide a systematic review on recent studies of IoT-based healthcare-monitoring systems through literature review. The literature review compares various systems’ effectiveness, efficiency, data protection, privacy, security, and monitoring. The paper also explores wireless- and wearable-sensor-based IoT monitoring systems and provides a classification of healthcare-monitoring sensors. We also elaborate, in detail, on the challenges and open issues regarding healthcare security and privacy, and QoS. Finally, suggestions and recommendations for IoT healthcare applications are laid down at the end of the study along with future directions related to various recent technology trends.

## 1. Introduction

The term Internet of Things (IoT) was invented by Kevin Ashton in 1999 and refers to data on the Internet that are connected to evolving global service architecture [1,2]. IoT is the product of advanced research on information and communications technology. It can potentially enhance urban residents’ quality of life. Since the global population is increasing at an astonishing rate, and the prevalence of chronic diseases is also on the rise, there is growing demand for designing cost-effective healthcare systems that can efficiently manage and provide a wide range of medical services while reducing overall expenses [3,4,5,6]. The IoT has become a key development area recently, enabling healthcare-monitoring system advancement. The IoT healthcare-monitoring system aims to accurately track people and connect various services and things in the world through the Internet to collect, share, monitor, store, and analyze the data generated by these things [7]. However, the IoT is a new paradigm where all connected physical objects in any intelligent application, such as smart city, smart home, and smart healthcare, are addressed and controlled remotely. Diagnosing disorders and monitoring patients is essential to providing medical care, and applying sensor networks to the human body will significantly assist in this endeavor. In addition, the information is readily accessible from any location in the world at any given time [8].

Patients with severe injuries or from certain areas may have difficulty reaching the hospital. Therefore, they can use video conferencing to communicate with their doctors to improve their health while saving money and time. Patients can use this technology to record their health conditions on their phones [9]. It is anticipated that the benefits of the IoT will be improved and result in individualized treatment, improving patient outcomes while saving healthcare management costs. IoT systems allow physicians to keep an eye on their patients remotely and schedule their appointments more efficiently. Patients also can improve their home healthcare to reduce their need for doctor visits and the likelihood of receiving unnecessary or inappropriate medical treatments in hospitals or clinics. For this reason, the quality of medical care and the overall safety of patients may improve, while the overall cost of care may decrease. The IoT holds significant potential in healthcare [7,10]. It will not be long before we have access to a health-monitoring system that can be used from the comfort of our homes and streamline hospital processes. IoT sensors should be densely deployed to monitor the body and environment continuously. This effort will enable the tracking of chronic-disease management and rehabilitation progress. In the future of virtual consultations for remote medical care, the IoT will be able to provide efficient data connections from multiple locations [11].

Most of the current implementations of the IoT and research on it are undeveloped and focus on deploying and configuring technology in various contexts and conditions. However, these practices are not widely used today. Therefore, this paper aims to evaluate related research on designing and implementing an IoT-based healthcare-monitoring system that improves quality of life. These systems rely heavily on IoT devices and sensors to connect patients with the healthcare providers best suited for their care.

The main contribution of this research paper is to highlight IoT-based healthcare-monitoring systems in detail so that future researchers, academicians, and scientists can easily find a roadmap to understand the current healthcare-monitoring systems and can easily provide solutions and enhancements for such critical applications. In this research paper, we provide a general idea of IoT-based healthcare-monitoring systems in a systematic way, along with their benefits and significance, and a literature review. Moreover, we discuss the concepts of wearable things in healthcare systems from an IoT perspective. The paper also provides a classification of healthcare-monitoring sensors, addresses security and protocols for IoT healthcare-monitoring systems, and details challenges and open issues. We also suggest solutions to overcome these challenges and issues in the future.

**Figure 1:**
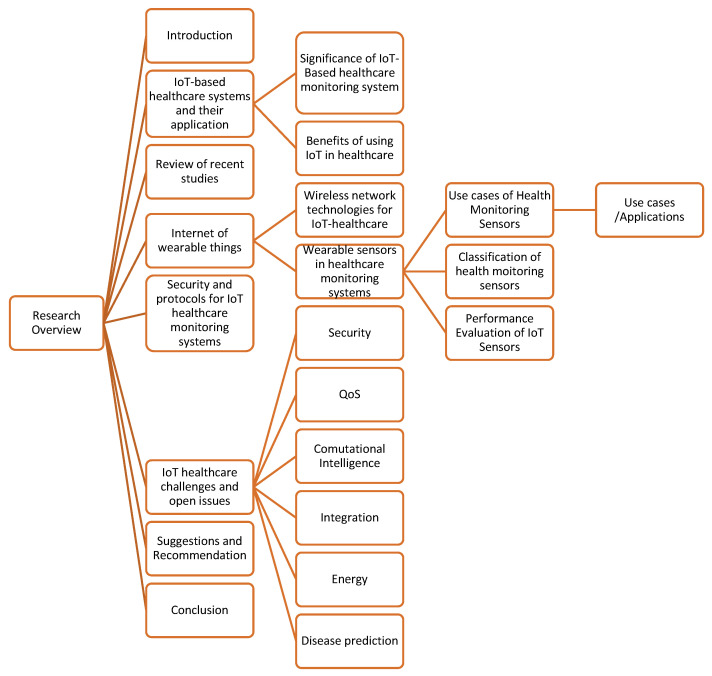
Research overview.

The paper is divided into eight sections as follows: Section 2 discusses the IoT-based healthcare system and its applications and the significance of using the IoT in the healthcare domain, followed by a review of the recent related studies in Section 3. Section 4 describes the Internet of wearable things and wearable sensors in the healthcare-monitoring system; this section also provides a classification of heath-monitoring sensors. Section 5 emphasizes security and protocols for IoT healthcare-monitoring systems. Section 6 describes IoT healthcare challenges and open issues. Suggestions and recommendations are described in Section 7, and Section 8 provides the conclusion of the overall review. Figure 1 shows the overall paper structure.

## 2. IoT-Based Healthcare Systems and Their Applications

IoT-based healthcare systems and their applications facilitates people’s lives in different ways, such as:

1. Remote healthcare: Wireless IoT-driven solutions bring healthcare to patients rather than the patient to healthcare. Data are collected securely through IoT-based sensors, and the data are analyzed by a small algorithm before being shared with health professionals for appropriate recommendations.
2. Real-time monitoring: IoT-driven non-invasive-monitoring sensors collect comprehensive psychological information. Gateways and cloud-based analysis manage the storage of data.
3. Preventive care: IoT healthcare systems use sensor data, which help with the early detection of emergencies and alerts family members. Machine learning for health-trend tracking and early anomaly detection is achieved through the IoT approach [12].

### 2.1. The Significance of IoT-Based Healthcare-Monitoring Systems

The development of monitoring systems for healthcare is receiving a great deal of attention from researchers and leaders in the medical field. Several successful research projects have been conducted in this area, and many more are currently underway [13]. The number of gaps in care provided by healthcare providers is increasing significantly, directly resulting from the rapidly growing number of older adults and patients with chronic illnesses. The major shortcoming is that healthcare is only provided in hospitals; therefore, it is unsuitable for seniors and people with disabilities and cannot always meet their needs [14]. The IoT, with the help of sensor values and telecommunications, provides an effective and practical solution to the issue of real-time monitoring of the health status of the elderly. It has been shown that the IoT, in conjunction with smart technologies, can provide various improved and enhanced services. Using sensors, researchers have developed various emergency systems using technologies that enable intelligent and remote wireless communication. These technologies have been used for various medical purposes, particularly in monitoring the health of the elderly. This way, data can be collected on general health and dangerous situations by capturing important vital signs [15].

### 2.2. Benefits of Using IoT in Healthcare

The IoT will reshape healthcare as we know it, with profound implications. In terms of how apps, devices, and people communicate with each other to deliver healthcare solutions, we have reached a whole new level of evolution. The IoT has given us a new perspective and tools for an integrated healthcare network, greatly improving healthcare quality.

The IoT has made it possible to automate healthcare procedures that previously required a significant amount of time and left room for error due to human involvement. For example, to control airflow and temperature in operating rooms, many hospitals now use networked devices.

There are almost endless ways the IoT can improve medical care; however, the following are some of the key benefits:

- Reduced cost of care.
- Human errors are reduced.
- Elimination of the limitations of distance.
- Reduced amounts of paperwork and record keeping.
- Chronic diseases are detected early.
- Improvements in medication management.
- The need for prompt medical care.
- Better treatment outcomes.

## 3. Review of Recent Related Studies

Healthcare is a vast arena that is composed of many different components. Delivering healthcare involves clinical practices, hospitals, pharmacies, home health providers, long-term care providers, pharmaceutical companies, and medical-device manufacturers. It also involves health and wellness products and services, insurance companies, and governments providing services to end-users [6]. This section provides a review with an analysis of the recent research on IoT-based healthcare-monitoring systems. Table 1 summarizes some of the recent studies regarding IoT-based healthcare-monitoring systems. 

The wearable device developed by Wu et al. [16] monitors various physiological parameters, including body temperature (BT), electrocardiograph (ECG), and heart rate (HR). Using Pulse Arrival Time (PAT) to measure ECG and PPG, it is possible to estimate blood pressure (BP). The interaction between humans and remote monitoring programs is straightforward because all the components are designed within a rigid framework. In addition, the power consumption of the devices is low, and they can communicate wirelessly to make tailored measurements of a specific physiological signal. The physiological measurements can be wirelessly transmitted to a gateway using a BLE module. The data are encrypted at the sensor patch and gateways to maintain privacy, ensuring transmission security. The wearable sensor system is connected to the cloud using a smartphone and a Raspberry Pi module as a gateway; the data can be retrieved and analyzed from the cloud. Despite its low energy consumption, BLE technology is unsuitable for wireless communication over long distances and high data rates.

Islam et al. [17] developed an intelligent monitoring system for use in a hospital. It not only collects data on patients’ BT, HR, and other vital signs but also monitors environmental factors in the hospital room, such as CO, CO_2_, and humidity. The success rate of modern healthcare systems is ~95% agreement between monitored and actual data in all cases. Medical staff can view the data in real-time, either on-site or remotely. Hypothetically, the technology would be helpful during medical crises and epidemics, as medical personnel would have almost instant access to raw data. The prototype created is incredibly easy to design and use. Such devices could be helpful in managing infectious-disease outbreaks, such as COVID-19. Potentially, this system could save more lives by improving the efficiency of the existing healthcare system. However, at this stage, the system still lacks some epidemic-related sensors that need to be evaluated once implemented.

In [18], Al-Sheik and Ameen propose an IoT health-monitoring system for cell phones that remotely monitors patients’ vital signs, including BT, ECG, and blood-oxygen saturation (SpO2). Arduino was used to measure and process this system. This system uses Wi-Fi to send the data to a cloud service on the IoT platform called Blynk; the data can be monitored in real-time. For security and privacy reasons, the results are sent to a specific smartphone that the doctor can monitor. Therefore, two microcontrollers, Arduino and NodeMCU, are used, which still need to be improved. For long-distance transmission, Wi-Fi technology is not the ideal option.

Hamim et al. [19] present an IoT-based healthcare-monitoring system for patients and older adults based on an Android application. The sensors in this prototype collect BT, HR, and Galvanic Skin Response (GSR) data that are fed into a single system, the Arduino Uno platform. Raspberry Pi transfers the data to cloud storage. Android Studio was used to develop the Android app, in which health parameters collected from patients can be visualized. Doctors can use the application to prescribe necessary prescriptions and track the patient’s health over time.

Using Raspberry Pi 3, Swaroop et al. [20] developed an IoT-based real-time health-monitoring system. Data creation, acquisition, processing, communication, and access are the main phases of the system structure. Health data such as HR, BT, and BP were measured. The data are transmitted through modes such as BLE, GSM, and Wi-Fi, i.e., mobile applications, messaging services, and the Internet. It was found that the latency is low, and there is no significant delay between sending and receiving data. Thus, the system’s accuracy is limited to the accuracy of the sensors.

Gupta outlines a healthcare-monitoring system using the IoT for obese patients [21]. The prototype is a fully functional device that measures body characteristics such as HR, SpO2, BP, and BT. This device is ideal for regular monitoring of body conditions. The system uses an Arduino board to store medical data for multiple patients simultaneously, and then, sends the information to healthcare providers via a Wi-Fi module for remote monitoring. Clinicians can use the recorded data to examine patients’ health patterns over time in order to detect any changes that may indicate an underlying, undetected health problem. Consequently, long-distance communication can be a challenge with this system. 

To help physicians diagnose and monitor their patients’ health status, Alamsyah and Ikhlayel developed a monitoring system based on an IoT that can detect vital signs [22]. The system uses sensors to collect vital signs such as HR, BP, and BT. The data from the sensors are gathered and processed by Raspberry Pi before being uploaded to the cloud. The data can be retrieved remotely through a mobile app that allows easy access for medical staff. The results of retrieving vital-sign data show that the instrument was developed and the system was tested and evaluated reasonably.

An IoT-based real-time health-monitoring system can save a patient’s life by continuously monitoring the patient’s vital signs. The real-time health-monitoring solution proposed by Sangeethalakshmi et al. [23] continuously monitors patients wirelessly via a mobile app and GSM. Sensors capture vital signs that are transmitted to the cloud via Wi-Fi. The system consists of a data-acquisition module, a microcontroller (ESP32), and software. This system regularly measures and stores the patient’s BP, BT, ECG, HR, and SpO2 and transmits the data to the physician’s cell phone for analysis. The system also includes an alert system in which the physician’s cell phone receives a message when the patient’s vital signs are outside acceptable parameters. However, the system is only a prototype that still needs to be evaluated, tested, and calibrated.

Another IoT-based vital-sign-monitoring system is described in [24] by Sahu et al. Similar to other systems, vital signs are monitored in real-time, and the data that are collected are locally stored, and then, transferred to the cloud, from where they can be evaluated. The system detects abnormalities, sends alerts, and calculates early-warning scores. By storing the data on a personal server, the Android app reduces the burden placed on central medical servers and minimizes the server’s maintenance costs. The system is compact, portable, and easy for patients to use. Additionally, the system has been tested and evaluated against most other systems in the field. 

A. D. Acharya and S. N. Patil designed and implemented an IoT-based smart medical kit for critical medical conditions [25]. This kit can provide a versatile connection to data from the IoT and can support emergency medical services such as intensive care units. The model collects, stores, analyzes, and distributes Big Data in real-time, enabling users to lower their health risks and reduce healthcare costs. This research aimed to reduce patient anxiety about regular doctor visits. With the help of this project proposal, patients’ and doctors’ time will be saved, allowing doctors to help patients in critical condition as much as possible.

Jennifer S. Raj [26] proposed a novel information-processing system for IoT-based healthcare-monitoring systems to manage Big Data in an IoT environment effectively. The entire data-processing process is divided into three stages: collection and aggregation, the classification and analysis of collected data, and decision-making. The experiments were conducted using Python. This model was experimentally verified in a simulation by using different health sensors. The parameters were compared with existing hierarchical clustering and backpropagation neural network models to validate the performance. This model leverages Apache Kafka and Hadoop to address the need for real-time data collection and offline processing. According to the authors of this study, the proposed method outperforms the more traditional hierarchical clustering model and the backpropagation neural network model in data processing and information extraction; they claim that their proposed model achieves 97% accuracy. The study does not provide a comparative analysis of time-efficiency for the model.

Kishor and Chakraborty designed a healthcare model using seven classifying algorithms [27]. Nine different disease related datasets were organized based on classifications. AUC, accuracy, sensitivity, and specificity were the four variables used to measure the classifiers’ performance. The three phases of this work were data collection, pre-processing and computation, and determining the results’ visibility to physicians or end-users, with the results stored on a cloud server. This study compared machine learning-based health models with previously developed work. Unlike other classifiers, the RF classifier has the highest accuracy, sensitivity, specificity, and AUC for a variety of common diseases, according to the study authors. This model can be extended for various purposes, such as weather forecasting, military, and food availability prediction.

In another study that is very similar to [27], Souri et al. suggested an IoT-based system for monitoring student health [28]. This model aimed to monitor students’ valuable metrics and identify behavioral and biological changes in students using cutting-edge student-support technologies. This approach consists of three levels: identifying the required data for the student using biological and behavioral factors, capturing the information using biosensors and intelligent IoT devices, and pre-processing the data. In this process, four classifiers were employed to assess the validity of the proposed model. The experiment results showed that the classification algorithms performed superbly in terms of precision, recall, accuracy, and F-score. The authors stated that SVM achieved the highest possible performance in predicting diseases in the proposed scenario. This system requires a local repository to reduce the time needed for emergency services, which saves bandwidth within the system. The response time of this system is not fast enough.

A healthcare system based on a Random Forest Classifier and the IoT was proposed by Kaur et al. [29] to improve interactions between patients and healthcare professionals. The experimental results were compiled using eight datasets on different diseases to determine whether the proposed work is successful or not. Five different machine learning approaches were used in this work. According to the authors, the Random Forest learning technique achieved a maximum accuracy of 97.26% when applied to the dermatology dataset. In addition, it was claimed that Random Forest provided good and accurate results for each dataset considered. Accuracy and area under the curve (AUC) were the two-performance metrics used for different machine learning techniques and datasets, respectively.

Gera et al. [6] concentrated on an IoT-based Cloud Talk platform-connected patient-health-monitoring system. This system streamlines the conventional workflow by providing all systems—including medical examinations, facilities, and tests—in one location. This system is capable of being implemented in a real-world setting because it consists of five fundamental components that are able to carry out a variety of tasks, such as collecting patient data from wearable IoT sensors, uploading the report to a cloud platform, analyzing the findings, and providing medical check-ups, diagnostics, and facilities to patients. In addition to these benefits, the system facilitates better decision-making and makes navigating the conventional workflow of the normal healthcare system simpler. In addition, it acts as a point of contact for the patient, the doctor, the pharmacist, and the diagnostician. There are restrictions on the system’s ability to manage patient healthcare.

SoonHyeong et al. [30] proposed an intelligent health-related monitoring system that detects abnormal movements such as falls based on sensor readings from accelerometers. After detecting abnormal movements, the system analyzes basic bio-signals such as a person’s BP, HR, and BT. Users, caregivers, and professionals can check that the patient has measured biometric data anytime, anywhere, using a smartphone. This monitoring system includes a JAVA-based Android service environment. The performance of this monitoring system was evaluated using datasets with information from fifty different individuals. In this model, blockchain technology is used to protect individuals’ medical data by increasing the data’s reliability while maintaining its confidentiality. With the help of a sensor chip, technology that is part of the IoT, the accumulation of personal medical information is stored and monitored in real-time. The transmission of sensitive medical data occurs in real-time via a mobile device only, such as a smartphone.

Piyush et al. [31] present a positive strategy for monitoring the daily life of Alzheimer’s patients and providing quality care to those affected by the disease. This work is based on data collected from sensors connected to the IoT that determine various parameters of the patient’s body, such as temperature, BP, pacing, and walking speed, to name a few. The Atmega microcontroller is used for collecting all this sensory data and information. All the collected information is transmitted to a cloud server using parallel communication to analyze the data. It is possible to retrieve the patient’s desired parameters, which helps provide real-time patient support. In addition, this work cannot predict the patient’s condition before the emergency becomes more serious.

An IoT-based healthcare-monitoring system with numerous sensors and an intelligent security system was presented by Hashim et al. in [32]. The system uses many sensors to collect vital signs such as humidity and room temperature using a DHT11 sensor, HR using a pulse sensor, and BT using an infrared thermometer. Data from the sensors that used the Arduino to gather information on the condition of the patient are sent to ThingSpeak and stored using the Wi-Fi module. The collected data are displayed on the LCD (cloud platform). When the sensor detects an abnormal reading, an SMS is sent to the smartphone using a GSM module to contact the patient’s family or doctor promptly. The performance of the temperature and pulse sensors was evaluated using various experiments. According to the authors, the percentage error of the infrared thermometer sensor is 1.2% lower than that of the current model. The user and physician can view the results when uploaded to ThingSpeak, but this system cannot monitor the patient remotely in real-time.

A platform for IoT-based health monitoring was proposed by Mostafa et al. [33] that uses a NodeMCU microcontroller to obtain readings from a DS18B20 temperature sensor and a Max30100 pulse oximeter to determine BT, HR, and SpO2 values. The readings are displayed on an LCD in front of the patient and on the Blynk app-enabled phones of the physician and everyone else involved. This project also included an infrared sensor (IR) that detects objects in front of it and activates a relay to pump disinfectant without being touched. According to the authors, the application takes only one minute, and their project works flawlessly compared to the conventional method. NodeMCU, a less-expensive and -complicated processor with built-in Wi-Fi, is used in this system, making it more cost-effective than other existing systems. Although the system is only for cardiac patients, it surpasses the traditional systems by providing a safer, easier, faster, and more affordable service.

A Wi-Fi-connected smartphone and an electronic wearable device ere used by Jenifer et al. [34] as part of an IoT-based health-monitoring system. This system uses sensors to measure the patient’s physiological parameters, including HR, BT, BP, and SpO2. The patient’s data are collected via Wi-Fi from a remote location and stored in a cloud server, and the health parameters are continuously monitored. If abnormalities occur, an automatic alert is sent to medical professionals with the patient’s location. However, this study does not include experimental data or a comparative analysis.

Dhruba et al. [35] use the IoT to monitor sleep apnea. They developed a simple application using a basic microcontroller and a selection of key health-related sensors. After analyzing data from five different people, the system provided results that were quite suitable for determining whether or not someone is suffering from sleep apnea. According to the authors, two people did not have any sleep apnea symptoms, although they had been diagnosed. The individuals in question were between 36 and 50 years old and had significant problems with their sleep patterns. The system successfully detected the presence of sleep apnea in these individuals. This system can also detect obstructive sleep apnea when a person is screened. In addition, a person with OSA is considered a patient if he or she is 50 years of age or older. With the help of this type of monitoring, people can detect sleep apnea at an earlier stage. People can learn more about sleep apnea and its detection with the help of this system. It will also help people solve any problems related to their ability to sleep. However, when the patient is sleeping, the worn devices may come off and cause an uncomfortable feeling.

Kshirsagar et al. [36] suggest an ongoing, low-effort electronic saline-monitoring and -control system that can automatically keep track of the rate of saline flow, the amount of time left, and the rate of infusion. This system can send data to the server from a distance and show the results, such as the saline bead rate, the condition of the failure, and how much time is left to empty the saline bottle, on the main screen. It can also show the volume of the mixture. However, this system only entertains a single purpose (electronic saline observation), and the contribution does not match the research title.

The research conducted by Tiwari et al. [37] focuses on developing an IoT-based remote monitoring system for healthcare using NodeMCU and the Arduino IDE. Ubidots is the IoT platform discussed in this article. The open-source IoT application is required for Ubidots to function correctly. It is also an application programming interface (API) that allows users to shop and retrieve data via HTTP and MQTT protocols while connected to the Internet or a local network. With this IoT device, it is possible to read pulse rate and measure temperature and BP. This configuration allows for round-the-clock monitoring of a patient’s vital signs and detects abnormalities that may be present. The results of the ECG test showed that the subjects’ average HRs were 72, 75, and 78 beats per minute, respectively. The recorded SpO2 percentages were as follows: 94, 97, and 98%, respectively. Finally, the participants in the experiment had a temperature of 94.78, 95.6, and 97.4 degrees Fahrenheit, respectively, when their temperature was taken. The authors noted that the design concept is simple and inexpensive to implement, considering its cost-effectiveness. On the other hand, transpiration could affect the design.

In order to monitor a person’s temperature, BP, HR, and SpO2, Vaneeta et al. [38] built a system based on the IoT. The ability of nearby clinics to communicate with city hospitals about their patients’ medical conditions makes this a valuable system for rural areas and small towns. The IoT system can alert the doctor or physician in case of any deviations from the standard values in the patient’s health. The maximum relative errors (percentage r) in the HR measurements, patient BT, and SPO2 were discovered to be 2.89%, 3.03%, and 1.05%, respectively. These values are comparable to the commercial health-monitoring system. This IoT-based health-monitoring device makes it effortless for physicians to collect real-time data. The system can monitor the parameters regularly because high-speed Internet is accessible. Additionally, the cloud platform enables data archiving, so those earlier measurements may be retrieved quickly. This system would make it possible to diagnose and treat COVID-19-specific patients early on.

Khan et al. [39] built an IoT-based health-monitoring system utilizing Arduino to measure a patient’s BT, HR, and SpO2. The data were then transmitted to an app using Bluetooth. The patient can gain a quick understanding of their current health status thanks to the information that is also transmitted to the LCD panel. COVID-19 patients, older adults patients, asthma patients, COPD patients, patients with chronic diseases, and diabetic patients can keep their condition under control with the assistance of this system over time.

The authors of [40] presented an ECG monitoring system for cardiac patients based on the IoT. The system is comprised of many components, including an ECG sensing network (data gathering), an IoT cloud (data transmission), and estimation results (data prediction). Based on the system, the authors operated Arduino Mega to process the received patients’ data and transfer them to the cloud using the Wi-Fi module in this system, ESP 8266. ECG information was stored in the cloud and was accessible using MQTT and HTTP servers. The linear regression approach determined the relationship between the properties of the ECG signal and the error rate. A prediction was performed to determine how much of a difference there was in PQRST regularity and whether it was enough for an ECG monitoring device. Acceptable outcomes have been attained by recognizing the quality-parameter values.

In this paper, we have discussed various healthcare-monitoring systems that are based on the IoT. These systems are very beneficial for both patients and medical professionals. Arduino, Raspberry Pi, NodeMCU, and Atmega are the four main embedded systems used to develop most existing health-monitoring systems. These embedded systems monitor patients’ health in real-time to ensure they receive the appropriate treatment on time. There are several research holes in the currently available IoT-based healthcare-monitoring systems. Most of the recent healthcare systems monitor HR, HR, SpO2, and BP. However, many other significant factors have not been considered, such as physiological, therapeutic, behavioral, and rehabilitation-related factors.

**Table 1:** Summary of the existing healthcare-monitoring systems based on IoT.

Authors with Reference	Aims and Contributions						Methodology	Hardware/Software Technology	Features	Evaluation Metrics	Protocol	Limitations
Gera et al. [6]	A patient health-monitoring system that is built on IoT technology and is connected to the Cloud Talk platform.	Used method known as software development life cycle (SDLC)	LM35, SEN-11574, MAX30102, and BMP 180.	Improves decision-making abilities and streamlines the normal flow of the healthcare system	Temperature, SpO2 level, BP, and pulse rate	IEE 802.11	Minimal contribution to the administration of medical care for patients					
Wu, Wu [16]	Developed a small wearable sensor patch that can assess a variety of physiological signals.						Uses a smartphone as the mobile gateway, Raspberry Pi 3 as a fixed gateway, and a BLE module for transmission parameters.	AD8232, PPG, and Si7051 sensors, RFD77101 and Raspberry Pi 3.	- The system is designed in a rigid-flex structure. - Measures ECG, HR, and BT and estimates BP via PAT. - Data are encrypted.	ECG, HR, BT, and BP.	MQTT	Range and bandwidth limitations.
Islam, Rahaman [17]	Proposed a real-time IoT system to monitor patients’ vital signs and the room’s environmental conditions.						Data from sensors are gathered, processed, and uploaded to the cloud using an ESP32.	LM35, Heartbeat Sensor Module, DHT11, MQ-9, MQ-135, and ESP32.	In cases of infectious disease, the system is helpful.	BT and HR, CO, CO_2_, and humidity.	HTTP	- The system appears to be big. - Need addition of some vital sensors to be useful in an epidemic.
Al-Sheikh and Ameen [18]	Designed an IoT healthcare-monitoring system that uses a mobile phone.						The system uses Arduino Uno to collect and process sensors’ data, followed by Wi-Fi transmission to the cloud.	Max30102, AD8232, LM35, NodeMCU, and Arduino.	- Developed as a mobile app. - Secures data by sending data to specific smartphone.	HR, SpO2, ECG, and BT	IEEE 802.11	- System uses two microcontrollers. - Wi-Fi range.
Hamim, Paul [19]	Developed a prototype of IoT-based remote health-monitoring system.						The system collects and processes sensor data using Arduino UNO and sends it to the cloud using Raspberry Pi 3.	LM35, HR Sensor Module, GSR sensor, Arduino, and Raspberry Pi 3.	- Designed as an Android app to access the Google Firebase database. - Data are encrypted and can be accessed by authorized personnel.	HR, BT, and GSR	IEEE 802.11	System uses two microcontrollers that make it quite big.
Swaroop, Chandu [20]	Enhances healthcare delivery by communicating multiplexed data over three modes—BLE, GSM, and Wi-Fi.						Monitoring three parameters and sending data using three modes.	DS18B20, Sunrom BP/ HR monitor, Raspberry Pi 3, BLE adaptor, and USB GSM module.	- The data can be accessed in multiple modes. - Sends alerts to caregivers without delay. - Reduces the risk of losing track of patient if one mode is interrupted or fails.	HR, BT, and BP.	MQTT, BLE CSR Mesh	Accuracy depends on the sensors.
Gupta, Parikh [21]	Designed a real-time IoT monitoring system to track and evaluate the health of obese adults. Can store the data of multiple patients.						The MCU includes a built-in keyboard, LCD, and all the linked sensors. The keypad gives the user access to the device’s menus and the LCD display. The data are gathered by the ESP8266 and uploaded to the cloud.	MAX30100, LM35, wrist BP and pulse rate monitor, Atmega 328, keypad, LCD, and ESP8266 Wi-Fi Module.	- The device is efficient, portable, and user-friendly. - The doctors and patients are alerted in abnormal situations.	BP, BT, pulse rate, and SpO2.	IEEE 802.11	- Uses two microcontrollers to process and transfer data. - For long-range application, Wi-Fi is not recommended.
Alamsyah, Ikhlayel [22]	Built an IoT-based system to monitor patients’ vital signs. Helps clinicians to make diagnoses.						This system uses Raspberry Pi for processing and communicating with the Internet using Wi-Fi technology.	MCP3008, HRM-2511E, DS18b20, MPX5050DP, and LCD.	Medical staff can access patients’ data through an Android device.	HR, BP, and BT	IEEE 802.11	Wi-Fi technology is not preferred for long-range application.
Sangeethalakshmi et al. [23]	Devised a real-time IoT-based system to track the condition of patients and save lives.						Detects vital parameters and sends them to ESP32 for processing and transferring to the cloud using Wi-Fi module.	LM35, AD8232, MAX30100, BP sensor, and ESP32.	- An application has been developed for doctors’ smartphones to monitor patients’ parameters. - An alert is sent to the doctor’s mobile phone in case abnormal readings.	Temperature, HR, ECG, BP and SpO2.	Wi-Fi/802.11	System needs to be evaluated, tested, and reorganized.
Sahu, Atulkar [24]	Created an IoT-enabled vital-sign-monitoring system.						Small electrical sensors are fitted to different bodily parts. Body sensor network transmits vital indicators to a controller via wireless or wired means (BSN).	ECG electrodes, pulse Oximeter, NIBP, BT sensors, STM32F103xC, CY8C58LP, and BLE 4.0 module.	System has an Android application and shows high accuracy measurements.	HR, SpO2, temperature, BP, and ECG.	Wi-Fi/802.11 BLE	Not suitable for long-range communication.
A. D. Acharya and S. N. Patil [25]	- IoT-based smart doctor kit. - Portable, simple, and low-cost kit.						The patient’s body has sensors attached. These send body data to the MCU; then, they send the data to the cloud via a Wi-Fi module.	AD8232, LM35, MPX10, Arduino, and Raspberry Pi Module.	- Designed as Android application for doctors and patients. - Monitors patients’ real-time condition.	ECG, temperature, and BP.	IEEE 802.11	Wi-Fi technology is not preferred for long-range application.
Jennifer S. Raj [26]	Innovative Big Data-processing platform for IoT-based healthcare-monitoring system.						Data processing is divided into three stages: collection and aggregation, classification and analysis of collected data, and decision-making.	- Data-accumulation and data-aggregation methods. - Classification and analysis of the collected data.	In comparison to the traditional model, it is efficient in the process of handling data and extracting information.	Data management, storage, f-measure, sensitivity, and specificity	Not provided	Data-processing time is not entertained.
Kishor and Chakraborty [27]	An approach to medical care that is underpinned by fog computing and makes use of AI and IoT						Three phases are involved. First, data are collected; then, they are pre-processed and computed; and lastly, the results are made visible to doctors or end-users and stored in the cloud.	- Seven machine learning classification algorithms. - Four performance metrics.	This model assists medical professionals in making accurate and timely diagnoses of the disease.	Heart disease, diabetes, breast cancer, hepatitis, liver disorder, dermatology, surgery data, and thyroid data.	Not provided	Predicts only the common diseases
Souri et al. [28]	A student healthcare-monitoring system based on the IoT.						This methodology has three levels: finding the relevant data, collecting the data, and pre-processing the data.	- Four data-mining algorithms. - Four performance metrics.	Utilizes innovative medical technologies and identifies changes.	Biological and behavioral changes.	Not provided	- Longer response time. - Limited for emergency services.
Kaur et al. [29]	Enhancing the interaction between patients and medical professionals						Eight datasets on different diseases were used to test the proposed work.	Five machine learning techniques.	Provides automatic recommendations.	Accuracy and area under the curve	Not provided	The performance comparison displayed here only includes accuracy and area under curve (AUC).
SoonHyeong et al. [30]	Enhanced reliability and security through the implementation of blockchain technology.						This study used blockchain-based IoT. Several sensors were used to assess ECG data.	Integrated sensor module	- User-friendly JAVA-based Android service environment. - Detects irregular movement.	BP, HR, temperature, weight, and ECG	BLE	Stored data/information can be transferred through smartphone only.
Piyush et al. [31]	Offers a mechanism for improving the quality of life of Alzheimer’s patients, and also benefits the people who care for them.						The study utilized IoT-based sensor data to determine various patient body parameters. All these sensors, attached to the MCU, are then transferred to the cloud.	LM35, pulse sensor, Gyroscope MPU6050, Atmega328 microcontroller, and ESP8266.	Dynamic estimation.	BT, BP, striding action, and speed.	IEEE 802.11	Cannot predict the condition of the patient before the situation becomes worse.
Hashim et al. [32]	Developed an IoT-based healthcare-monitoring system with multiple sensors and a smart security system.						Multiple sensors are connected to Arduino, and the collected data are presented on an LCD. The Wi-Fi module transmits data to the cloud.	DHT11, pulse sensor, mlx 90164, Arduino, LCD, and ESP8266 Wi-Fi module.	- Sensors are wearable and safe. - SMS.	HR, BT, room temperature, and humidity.	IEEE 802.11	The size of the prototype needs to be reduced and enhanced.
Mostafa et al. [33]	Designed an IoT that can monitor patients’ readings continuously; keeps the data on display in front of the patient and on the screen of the doctor’s mobile device.						Three sensors are read by MCU with availability to represent the data locally and remotely.	Max30100, DS18B20, IR sensor, NodeMCU, and LCD.	- Cost-effective system. - Automatic Hand Sanitizer. - Short time to perform all processes.	HR, SpO2, and temperature	Wi-Fi/802.11	The prototype’s size should be minimized.
Jenifer et al. [34]	Designed an IoT based on electronic sensors to monitor patient healthcare remotely.						Sensors collect data on various physical factors and upload them to the cloud database over Wi-Fi.	LM35, Arduino Uno, SIM300, GPS shield.	Automatic emergency alert message and location can be sent.	HR, temperature, BP, and SpO2 level	IEEE 802.11	- No comparison with the existing systems is provided. - Experimental data are not presented.
Dhruba et al. [35]	Developed a real-time sleep apnea-monitoring system based on the IoT.						Takes readings of sensors and measures several sleep indices, and alerts users via a mobile application when anything unusual occurs.	Max 30102, pulse sensor, GSR sensor, AD8232 and sound sensor, Arduino Uno, and Bluetooth module.	- Mobile application provided. - Diagnoses sleep apnea in earliest stages.	GSR, ECG, HR, sound, and SpO2.	BLE	During sleep, the worn device can be detached and feel uncomfortable to the patient.
Tiwari et al. [37]	Designed a system for remote monitoring of healthcare based on IoT.						Performs ongoing observation of a patient’s vital signs and detects the presence of abnormalities.	LM35, MAX30100, AD8232 and IR sensors, NodeMCU, and Arduino IDE.	Simple to operate and affordable due to its high level of cost effectiveness.	HR, temperature, and ECG.	MQTT, HTTP IEEE 802.11	- Number of subjects is very low. - Product may be affected by sweating.
Vaneeta et al. [38]	Conceived and built an intelligent health-monitoring system based on the IoT.						Consists of three primary steps: data collection, data processing, data storage, and the display of patients’ parameters locally and remotely.	MLX90614 and MAX30100 sensors, BP serial port, LCD, and Raspberry Pi.	This system will send an alert to the attending doctor or physician if there have been any deviations from the normal values of the patient’s health.	BP, HR, SpO2, and temperature.	IEEE 802.11	Need to increase the security of patients’ data and decrease the data-transfer delay.
Khan et al. [39]	Established a mechanism for measuring multiple health indicators quickly.						Sensors capture information on various physical factors and upload them to the cloud using the Bluetooth module.	LM35, MAX30100, Arduino UNO, Bluetooth module, and LCD.	Data can be monitored using mobile app.	BT, HR, and SpO2.	BLE	The size of prototype needs to be enhanced.

Note: Aims and Contributions refers to the aim of the research work. Methodology refers to methods and techniques used. Hardware/Software Technology refers to hardware and software used. Features refers to main features addressed. Evaluation Metrics refers to evaluation metrics. Protocol refers to protocol utilized. Limitations refers to drawbacks of research works.

This may be another serious and urgent situation that a sophisticated health-monitoring system needs to consider, ensuring that prompt assistance and medical support are provided. Because the monitoring is performed in real-time, specific existing systems are deficient in their ability to aggregate data from the monitoring device. These data should be stored in the cloud to be analyzed later to determine whether or not an emergency exists in a patient’s profile. The differences and similarities between various IoT-based health-monitoring systems are outlined in Table 1.

The gaps in the existing system can be summarized as follows:

1. The IoT has the potential to be integrated with a wide variety of devices, which is not possible with most of the systems that are currently in use.
2. There is the possibility that the data that are stored will not be protected.
3. Complex systems have many disconnects between the various people, stages, and procedures.
4. An investigation into the circumstances surrounding an accident will typically reveal the existence of several gaps, but gaps themselves are rarely the cause of accidents.
5. The ability to understand and reinforce the normal ability of practitioners in order to bridge gaps contributes to an increase in overall safety.
6. The conventional viewpoint, which maintains that systems ought to be shielded from the unreliable influence of humans, is challenged by this point of view.
7. We have a limited understanding of how professionals pinpoint newly formed gaps and devise solutions to close them when systems undergo transformation.

## 4. Internet of Wearable Things

The Internet of Wearable Things (IoWT) aims to improve people’s quality of daily life. It involves sensors fitted into wearable devices, monitoring the individual’s activity, health factors, and other things. The data collected from the IoWT can be fed into medical infrastructure, giving clinicians remote access to their patients’ data as they go about their daily lives. Building on the IoT architecture, a novel integrative framework for IoWT is currently being developed. The IoWT is a revolutionary technology that has the potential to change the healthcare industry by creating an ecosystem for automated telehealth treatments [41].

**Figure 2:**
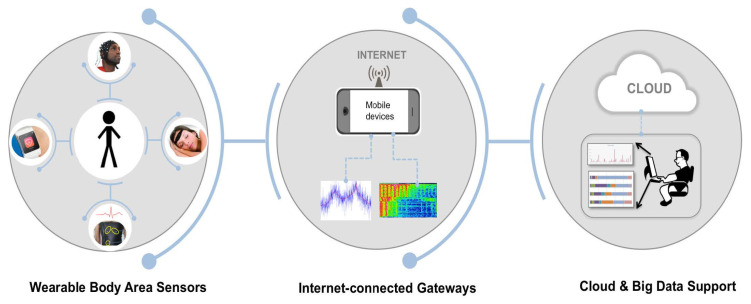
Architectural elements of IoWT [42].

As shown in Figure 2, the architecture of the IoWT and its connections consists of three elements: the WBAN, the gateway connected to the Internet, and the cloud. The WBAN is a front-end component of IoWT that wraps around the body to collect health-related data unnoticed. The WBAN collects data from sensors in direct contact with the body or from sensors in the environment that can collect indirect data about a person’s behavior. The WBAN can either analyze the data or transmit them for remote analysis. In addition, mobile computing devices such as smartphones, tablets, and laptops must be connected to the Internet to send data to powerful computing resources [42].

### 4.1. Wireless Network Technologies for IoT Healthcare

Healthcare systems can be monitored remotely using various wireless network technologies. The existence and operation of IoT emerging technologies, such as RFID, wireless network technologies (BLE, Wi-Fi, Zigbee), and low-power wireless area network (LPWAN) technologies (such as LoRa and SigFox) are engaging in terms of the IoT’s long-term development and deployment. They enhance device connectivity to the Internet, and the efficiency of IoT application operation [43].

BLE, LoRa, and Zigbee are wireless sensor network technologies; meanwhile, to identify and trace products, RFID is used. BLE can transfer data between different mobile devices [44]. Communication methods can be long in their range (LoRa, SigFox, and Wi-Fi) or short-range (Bluetooth, RFID, and Zigbee) [24]. Due to new communication protocols being created exclusively for IoT devices, such as LoraWAN, NB-IoT, and Sigfox, it is anticipated that the popularity of these applications will increase, enabling a far-reaching remote monitoring system [11,45].

**Figure 3:**
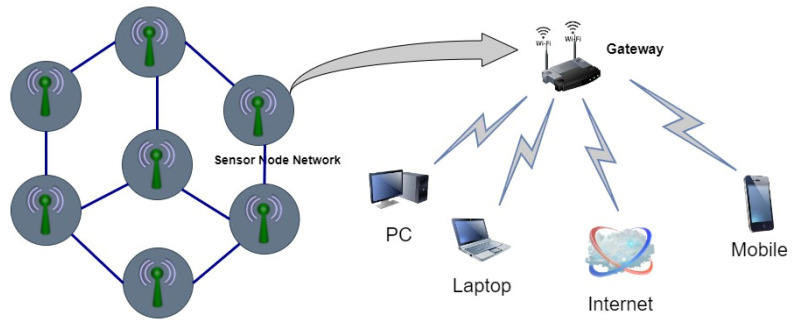
Relationship of WSN to IoT [46].

An essential component of the IoT is the WSN. The IoT, which has already been established, can connect things to the Internet, allowing humans to interact with computers and for computers to interact with other computers. Thus, the combination of the IoT and WSN facilitates machine-to-machine communication. Figure 3 illustrates the architecture of IoT with the WSN. It shows sensor nodes communicating with a gateway in a separate network. Many devices are linked to the gateway via Wi-Fi or the Internet, ensuring interoperability [46].

The researchers in [24] counted the existing wireless applications in connected healthcare facilities to study operational wireless methods for transmitting data across short distances. The system design and implementation of family mobile medical care are presented in this study. The Android mobile client, data transmission, and a system server are part of the system. Wireless data transfer is potentially possible, at least in theory. An example of the mobile healthcare system’s success is shown here. In the first place, family members’ sign characteristics might be collected via sensors on medical equipment. ECG, BP, SpO2, respiration, and sleep are parameters of interest. The mobile terminal uploads data to a back-end Web server with a wireless network, Bluetooth, and Wi-Fi. Data storage, computation, and analysis are all handled by the MySQL database server [24]. A family member’s smartphone or tablet may be used to show data icons or text, making it easy for them to monitor their loved one’s health at any time and location. Family members may prevent significant health issues through early intervention, encouragement, and healthcare maintenance.

### 4.2. Wearable Sensors in Healthcare-Monitoring Systems

In real-time, the healthcare sector may use wearable devices to monitor and save patients’ activity and physiological functions. Such devices have one or more sensor nodes, but each sensor node typically has a radio transceiver, a low-speed processing unit, and small memory. The sensors can measure various physiological parameters and activity, including SpO2, BP and temperature, electrodermal activity (EDA), ECG, electromyography, HR, and RR [2,47].

Bluetooth, infrared, near-field communication (NFC), RFID, Wi-Fi, and Zigbee wireless transceiver technologies can support wearable devices communicating with smartphones and other devices. The technology promotes care by facilitating remote diagnosis and monitoring [11]. An important issue of discussion in this period revolves around the IoT in healthcare. One of the essential parts of healthcare is identifying and treating illness. In order to achieve this, the body sensor network will be valuable. Additionally, the data may be accessible from any location in the world [8].

A wearable sensor gadget created by Vedaei can monitor and analyze the actions of patients. An IoT technology that measures social distance might help prevent a COVID-19 sufferer from becoming sick. Three layers of IoT sensors, machine learning algorithms, and smartphone apps are used to monitor BP, SpO2, cough rate, and temperature daily. The frameworks outlined by the authors helped the users keep a safe distance between themselves and the transmission of the virus and update their information often. A distance-monitoring system based on Radio Frequency (RF) was also presented in the research, which may be used in both indoor and outdoor contexts. In order to compare the findings under environmental restrictions, the authors looked at two alternative situations. Those who wrote the article claim to have helped expose COVID-19 [48].

**Figure 4:**
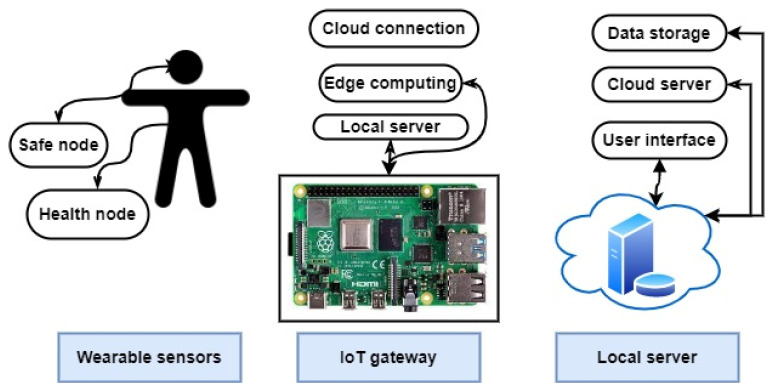
Healthcare-monitoring system using wearable sensor [49].

Another study [49] demonstrated an IoT-connected wearable sensor network system for industrial outdoor workplace health and safety applications. Wearable sensors worn by the worker collect physiological and environmental data, which are transferred to the system operator and employees for monitoring and analysis. Data harvested from multiple workers wearing wearable sensors can be transferred through a LoRa network to a gateway. The LoRa network combines a Bluetooth-based medical signal-detecting network with a heterogeneous IoT platform. The authors describe the sensor node hardware and design, the gateway, and the cloud application. A heterogeneous wearable IoT device sensor network system for health and safety usage is shown in Figure 4.

#### 4.2.1. Use Cases of Health-Monitoring Sensors

Medical science research is currently dominated by medical healthcare, which mostly relies on how it integrates with the IoT. This integration is receiving a lot of attention due to its crucial role in utilizing technological paradigms to save human lives. These integrated systems contain three crucial phases, namely, the modules for data collection, data processing, and data evaluation. Healthcare monitoring plays a significant role in the data collection module due to its active involvement in gathering data from various sources and specimens. Most healthcare-monitoring systems use sensors to obtain the necessary input data. The more concise and timely the data, the more accurate the results.

Sensors are employed for more than just data collection; they can also be used for various ongoing and post-monitoring tasks in IoT-based healthcare systems. Blood pressure, body temperature, pulse oximetry, and blood glucose are a few examples of heterogeneous wearable sensing devices developed to collect patients’ biomedical data [50] in the era of fast-growing IoT. The proper quality and development of these IoT-based healthcare-monitoring systems are directly related to reliable data from sensors or sensor networks, which necessitates using advanced signal-processing techniques, sensor data fusion, and data analytics. In medical science, sensors that measure heart rate, body temperature, and other things are used to find and diagnose diseases at the earliest stage.

It has been observed that health-monitoring sensors are utilized in various use cases of medical science for healthcare purposes, such as the monitoring of hemoglobin concentration, molecular diagnostics, clinical diagnosis of albumin-related diseases, heart-rate detection, blood-oxygen-saturation detection, respiratory-rate detection, anemia detection, Alzheimer’s disease, and many more.

There are many applications for wearable sensors. IoT-assisted wearables are widely used these days. The friendliness of such devices has created a boom in their application in all fields. With the healthcare field being no exception, the IoT’s exploits in healthcare are enormous. Various technologies are linked to existing technology that helps generate data for monitoring and analysis.

We have seen a lot of use cases for IoT-based sensors in real-time environments, which are mentioned below:

#### Use Cases/Applications

1. Heart-rate detection/Cardiac monitoring systems/Stroke

The first application of health-monitoring sensors was through IoT-based healthcare-monitoring systems; these can gather and measure the necessary data, transmit these data through various stages reliably to the gateway and the cloud server, and perform some edge tasks to provide low-latency decision-making for cardiac-related diseases and prediction. Some of the pieces utilize sensors to determine heart rate [51]. Several projects involve using WSN technology to continuously monitor heart patients who need a real-time monitoring system [52]. This WSN has several medical-grade sensors and devices that can track blood pressure, body temperature, heart rate, and pulse. A critical patient’s real-time ECG is also preserved so that the patient is continuously watched [52,53,54,55,56].

2. Body-temperature measuring

During the pandemic, IoT-based smart health-monitoring devices with sensors for COVID-19 patients based on body temperature, pulse, and SpO2 were beneficial. Through a mobile application, these systems can measure a human’s body temperature, oxygen saturation, and pulse rate [57].

3. Activity recognition

One of the many uses for medical wearables now being used is activity recognition. Almost all fitness trackers perform this kind of recognition. Fitness trackers are now the most popular wearables for tracking a person’s activity. A lot of guesswork is being carried out in the background, but most of them include a highly sensitive 3D accelerometer that allows the sensor to determine the acceleration [51].

4. Blood-glucose monitoring and hemoglobin concentration

Heart-rate sensors, blood-glucose monitors, endoscopic capsules, and other devices make up the Internet of Medical Things (IoMT), which together, create the IoMT diabetic-based WBSN monitoring system [58,59].

5. Respiration-rate detection and monitoring

We can keep an eye on the human body’s respiratory system in several ways. Some writers employed sophisticated sensors that keep track of breathing patterns. A bio-impedance sensor can be useful [51,60,61].

6. Sleep monitoring

This sleep-tracking app assists the user in adjusting their sleep patterns and maintaining a healthy life cycle. For this, various sensors are utilized. Wearables often track heart rate, pulse rate, SpO2 levels, and breathing patterns, and by taking these measurements into account, they may make an educated decision regarding the quality of sleep [62].

7. Alzheimer’s disease monitoring and Anemia detection

Monitoring for Alzheimer’s disease has several issues and needs to be handled carefully. When a patient is alone, diagnosing them with Alzheimer’s is impossible [63,64,65,66].

8. Molecular diagnostics and Clinical diagnosis

Due to quick and affordable healthcare applications with reduced risk of infection, recent developments in biosensors for patient-friendly diagnosis and implantable devices for patient-friendly therapy have attracted a lot of attention. The rapid development of point-of-care (POC) sensor platforms and implantable devices with specialized functionality has been made possible by incorporating recently created materials into medical equipment [67,68]. A lot of work has been conducted on the clinical diagnosis of albumin-related diseases [69,70].

9. Blood-oxygen-saturation detection

Along with precise, ongoing monitoring of intravascular oxygen levels, it is crucial to monitor patients’ cardiovascular health following cardiothoracic surgery [71]. There are new types of data, such as oxygen saturation, which are continuously collected using oxygen-saturation (SpO2) sensors and represent the percentage of oxygen-saturated hemoglobin compared to the total amount of hemoglobin in the blood; these are becoming available for market wearables. Other behavioral and physiological biometric types are already available in many market wearables [72,73,74].

Thus, it has been shown that health-monitoring sensors are used in various applications and can be used in the future for various diseases, particularly those that focus more on sample or data collection, monitoring, or evaluation. We may assert that whenever a sensor is employed, there is a possibility to collect the necessary data and deliver the desired outcomes, depending on precision and accuracy. Additionally, incorporating the cloud, geographic information systems, and mobile devices has improved the process of sensor-based data gathering and monitoring while allowing for flexible remote sharing and communication.

Numerous case studies and applications are possible for health-monitoring sensors. They can be used to measure hemoglobin concentration; for molecular diagnostics; to provide clinical diagnoses of disorders associated with albumin; to measure heart rate, blood-oxygen saturation, respiration rate, and anemia; to diagnose Alzheimer’s; and for many other things.

#### 4.2.2. Classification of Health-Monitoring Sensors

With advancements in wireless communications, medical sensor technology, and data-collection methods, it is now possible to remotely monitor a person’s health by putting wearable technology on them and analyzing the data collected. These sensors and wearable devices can be integrated into various accessories such as clothing, wristbands, glasses, socks, hats, and shoes, as well as other devices such as smartphones, headphones, and wristwatches.

**Figure 5:**
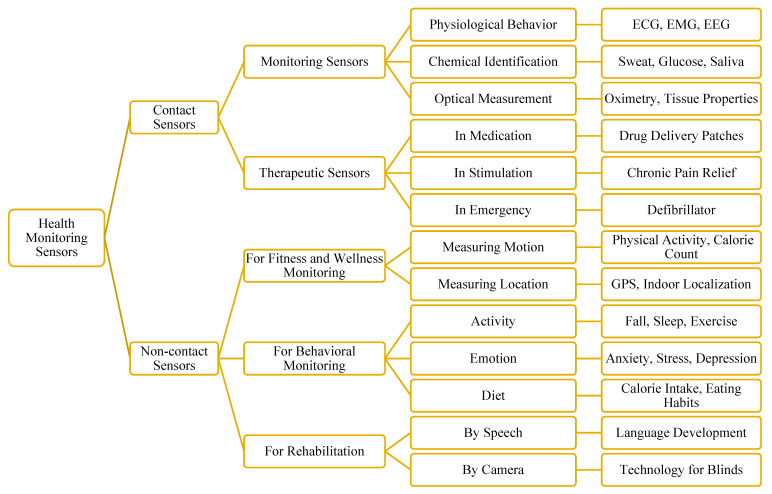
Classification of health-monitoring sensors [75].

Pawan Singh [75] classified medical sensors into two categories: contact sensors (i.e., on-body or wearables) and non-contact sensors (i.e., peripherals). Contact sensors are further classified into two sub-categories: monitoring and therapeutic. Again, non-contact sensors are further classified into three sub-categories. All the sub-categories are further classified based on their use. Figure 5 illustrates the classification of health-monitoring sensors with examples of their use. 

Primarily, health-monitoring sensors can be divided into contact (i.e., on-body) and non-contact (i.e., peripheral) sensors. Contact sensors are attached to the body to monitor physiological behaviors, chemical-level identification, and optical measurement-related monitoring. Contact sensors are also used in therapy-related monitoring such as medication, stimulation, and emergencies. Non-contact sensors are used for monitoring fitness- and wellness-related factors, behavior, and rehabilitation. An example of each type of monitoring is shown in Figure 5.

The following are some of the medical applications that could benefit from the use of medical sensors and wearable devices [76]:

- Monitoring vital signs in hospitals.
- Aging in place and in motion.
- Assistance with motor and sensory impairments.
- Large-scale medical and behavioral research in the field.

**Figure 6:**
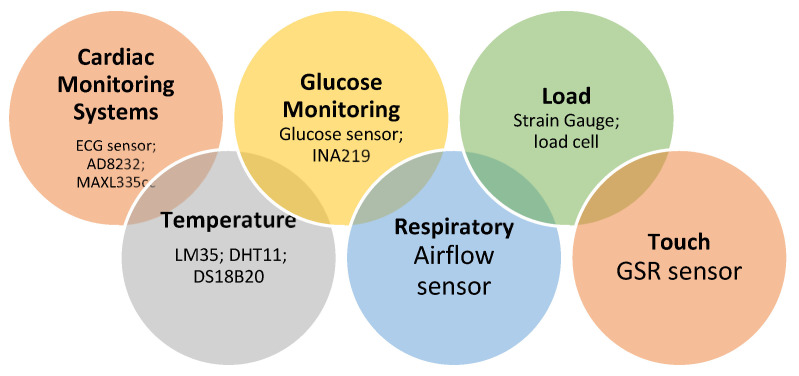
Various application of use cases and IoT sensors for healthcare monitoring [62,77,78,79,80,81,82].

Based on the applications in which they are most frequently used, we have divided health-monitoring sensors into different groups for performance-wise evaluations. These sensors can be divided into many categories, which are covered in the subsections. Figure 6 is a collection of several wearable sensors applied in various research projects and employed in IoT systems in healthcare.

#### 4.2.3. Performance Evaluation of IoT Sensors

Any healthcare-monitoring system’s sensors serve as its brain and heart. Thus, they must be reliable. Almost all types of sensors used should be small, quiet, accurate, have short data-transmission delays, use little power, and perform well overall. Wearable sensors must be both precise and compact, which presents a challenge. However, in case of wearable sensors, the more value is given to outputs, and they need to be reasonably accurate, too, so that the doctor can use these values to make decisions. Medical-grade sensors are large and difficult to transport and require specialized equipment and trained personnel [51].

Additionally, various IoT sensor-based applications constantly require authentication, security, and privacy. Numerous protocols are readily available on the market to assist with security and help offer some solutions over an extended period. Nevertheless, these integrated and crucial data-based apps’ security measures are constantly vulnerable to intrusion.

## 5. Security and Protocols for IoT Healthcare-Monitoring Systems

Along with the utilization of the IoT, there has also been an increase in the risk of new security assaults and weaknesses in healthcare systems. Healthcare data are highly sensitive and contain personal identifying information such as social security numbers. This is because many medical devices collect and share critical and sensitive patient-related data on the Internet via various connected devices for further evaluations and decisions. IoT technology’s nature presents complexity and incompatibility difficulties in medical-related IoT devices [83]. As a result, security issues such as a lack of availability, confidentiality, and integrity arise. Some of the IoT healthcare solutions include software and hardware that monitor and regulate patients’ vital signs in the form of monitoring services, which are connected to the IoT for data processing. However, these solutions are always at a high risk of security threats such as authorization, privacy, and authentication breaches [84]. Cybersecurity in healthcare has emerged as a big problem. Device flaws could be exploited by hackers, resulting in IoT system operational disruption. More importantly, due to the limitations of medical equipment, such as their scalability, power consumption, and interoperability, standard security criteria for countermeasures for attacks are not relevant. Moreover, when it comes to criteria for security, privacy, and dependability, the medical IoT technology should be trusted too. Additionally, some physical and technical protections to prevent data leakage are available on the market. However, these measures have fallen short of what is needed; stronger and more modern security standards should be implemented, and a resilient strategy should be implemented to save the crucial data [85,86]. Therefore, to better understand and develop a secure IoT-based healthcare infrastructure, it is necessary to also determine security requirements [87].

The available solutions could include more secure overlay networks such as the Onion Router (TOR) network, which might be used to transfer confidential data. Moreover, authentication and identity-verification methods such as signatures, voice patterns, finger-print scanning, passwords, and smart cards could be employed in application protocols. Existing security solutions, such as RSA, seed phrases, and DSS, may also be used at all connection endpoints. Technologies such as SDN, blockchain, and NFT tickets could be used to provide authentic and customized service. Last but not the least, artificial intelligence-based approaches that can be used to detect anomalies in IoT networks [87,88] could be implemented to overcome the issues and challenges of security in IoT-based healthcare-monitoring systems. 

Eventually, with the advancements in the IoT’s common standards, many protocols have been created to evaluate the services that are used for IoT solutions, and their relevance, to connect a variety of devices to the Internet and various architectures. IoT protocols for a particular application are selected considering the application’s requirements [89,90]. Wearable technology, smart medical equipment, smart homes, and remote monitoring are some of the IoT’s most exciting healthcare applications. Some recent studies emphasized IoT interoperability, which includes the healthcare-domain aspects of the IoT, which should compulsorily include the standardization of dependable communication protocols for improved and enhanced mobile and wearable technology. In addition, low-cost, low-power embedded processors are useful solutions. The most popular emerging IoT communication protocols that are extensively used to develop smart IoT applications include CoAP, MQTT, XMPP, AMQP, DDS, LoWPAN, BLE, and Zigbee. The most promising IoT-based healthcare apps for patient monitoring, therapy, and diagnosis are dependent on these protocols. The main uses of these protocols are to enhance the performance of telehealth, medication management, chronic-disease detection, bio-physical parameter monitoring, home and eldercare, and chronic-disease monitoring [88].

## 6. IoT Healthcare Challenges and Open Issues

**Figure 7:**
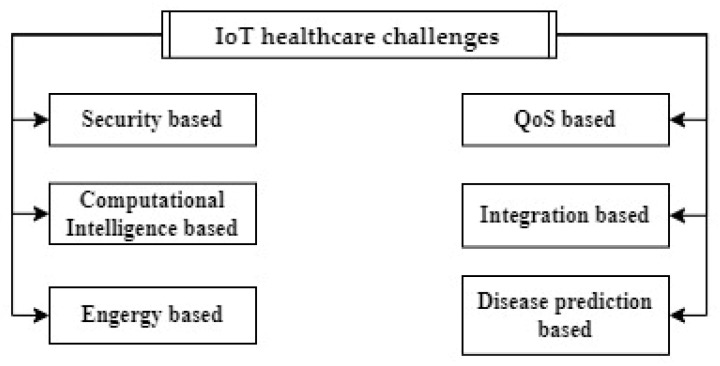
IoT healthcare challenges and open issues.

Although the IoT can provide personal health benefits, building data-collecting schemes that are efficient and secure to use in IoT healthcare-monitoring systems still presents numerous limiting issues. These various open research challenges, which include functionality, performance, data privacy, reliability, security, and stability, are considered in this section. We have divided the challenges and open issues into various categories: security-based, performance-based, computational-intelligence-based, integration-based, energy-based, and disease-prediction-based (see Figure 7).

### 6.1. Security-Based: Security and Privacy

There are ethical challenges to privacy and security. Hackers can easily access medical records, which are transformed into digital records (stored in electronic health records) and stored in the cloud. In a security breach of the cloud server, hackers can access patients’ medical data. This causes problems with user authentication, data ownership, data-protection policies, and misuse of health information [21,30,91].

Security and privacy must be addressed in IoT system design and development to improve confidence in employing the IoT in healthcare. Each IoT layer and component must have security protocols to reduce security risks and protect privacy. Developers must ensure that IoT “things” and the systems they connect to are secure, so that users can rely on sensors, devices, gateways, and IoT services, and so that their identity, safety, and privacy are protected. Numerous commercial and personal technologies are built without ensuring these security and privacy factors [92]. Various health IoT-based remote monitoring applications are majorly influenced by the integrated security mechanisms with built in hardware and software components of sensors and transceivers for wireless communications. Applications that collect data from these sensors and devices are typically designed with privacy in mind, using strong authentication and encryption measures and other safeguards, during both storage and transmission. Commonly, these apps integrate with pre-existing healthcare information providers, whose own security measures and privacy policies are put into effect. It is still possible that they have not adopted the most recent measures to secure data [11,93].

Solutions aimed at protecting individuals’ privacy should give people the power to choose who can lawfully view and make changes to their data. Users of the IoT need to trust that their personal information will be handled securely and responsibly. Multiple laws and policies, such as HIPAA and the EU’s General Data Protection Regulation, have already addressed privacy concerns when creating IoT applications (GDPR). There is, nevertheless, a requirement to think about the secondary use of the data gathered via home IoT remote monitoring. Patients using these systems may provide their permission for their information to be used just for the home health-monitoring system [11,94].

Indeed, securing data and assuring privacy remains a key challenge to the health IoT. Data that are transmitted to the data-processing unit could be spied upon, or the data could be manipulated, leading to a flawed analysis of Big Data. Therefore, ensuring the data are transmitted securely from the nodes to the processing unit is critical. Furthermore, during data processing, the identity of the individual yielding the data must be protected. By adopting cryptographic methods, the algorithms that process the data do not need to map the data to the user [91,95,96].

### 6.2. QoS-Based: Performance, Fuctional Stability and Reliability, and Cost

The priority of Quality of Service (QoS) is not consistent but varies depending upon needs; it safeguards a particular level of data-transmission performance. The primary challenge is maintaining the integrity of sensitive patient data while exchanging data from the end node to the server node. In the IoT, latency is the duration needed to send a packet of data between node devices [97].

QoS indicators apply to all the IoT architecture sub-components, from the home of the individual to the healthcare cloud services. Memory consumption needs to be checked to ensure there is no leakage of memory or data being cached inappropriately. Delays and interruptions to data transfer due to wireless disruption result in unexpected disconnection or erratic connectivity, poor signal, and slow network speeds. Another performance metric is energy-consumption management, which can also lead to reduced functionality, reliability, performance, and stability. The process of continuously collecting data is energy expensive for the devices. Following a period of battery discharge, the battery needs a period in which to recharge, but during that period, the device is unable to monitor continuously. When the charge of the battery is low, the device can experience a symptom comparable to wireless interference [11]. Where there is insufficient power for the wireless sensor nodes to operate, a severe issue is presented. To enable the nodes to function at low power, more effort should be dedicated to devising energy-efficient solutions, renewable technology, and green energy [8].

The IoT offers flexibility for monitoring patients who require ongoing medical evaluation, allowing the patient to live at home rather than being in the hospital. However, some patients find wearable devices to be uncomfortable. The data can become noisy as they are first transmitted from the sensor to the control device, and then, forwarded to the monitoring center. With superior architecture, data can be shared with minimal loss of integrity. Data signals can also be enhanced by applying noise-removal techniques. Most methods currently used to monitor ECGs require the signal to be analyzed in a supervised manner, which makes the process more expensive and can result in detection errors. To reduce costs and improve efficiency, machine learning can be used to analyze signals [98].

Another parameter is the cost of medical services, and treatment equipment is more important than ever. Researchers need to discuss and put more effort into minimizing the costs associated with IoT healthcare systems. The high cost of monitoring equipment in the IoT healthcare system is a serious issue. IoT has not yet made treatment services accessible to the average individual at a reasonable price. The cost of medical equipment is increasing [99,100].

To improve users’ perceptions and experiences of such expensive devices, it is incumbent upon device developers, manufacturers, assessors and testers to address these issues without compromising on cost or quality [11]. If the challenges outlined above can be resolved, the future IoT in the healthcare sector will be improved.

### 6.3. Computational Intelligence-Based

Computational-intelligence technologies are still in their infancy. Advanced intelligent computational services are needed for IoT-based healthcare-monitoring systems since computational intelligence is always a backbone of healthcare; it is associated with Internet-related data collection, computations, and evaluation because computing in IoT-based healthcare-monitoring systems is performed on edge devices to optimize data, networks, and traffic accordingly. However, we cannot ignore the fact that edge devices have limited resources and processing power, so we cannot ignore their limitations [11,101].

### 6.4. Integration-Based

Integration refers to the connection of current devices or tools with external technology to ensure the accuracy and consistency of data over the course of their lifetime for future expansion. The integrity of the data is still plagued by unresolved problems. IoT-based monitoring systems, when extended and fused with other external device that have various advantages, will improve quality of life. The development of integrated tools will have a significant positive impact on the communications, processing, and services provided by integrated information systems. This means that the IoT healthcare-monitoring systems needs to be extended using various technologies or related technologies such as the cloud, SDN, etc. [11,102]. 

### 6.5. Energy-Based

Monitoring-based healthcare-related IoT devices have a limited battery life. These gadgets still use energy even when they are in energy-saving mode and are not expressly required to read sensors. Some functions must be performed even when the device is in energy-saving mode, but it has a power limitation. Many pieces of medical equipment always need batteries, especially wearables and equipment for patients who need continuous condition monitoring [99]. An ideal system that integrates low-power communications with a power-efficient hardware architecture is needed to allow prolonged monitoring. Reduced power consumption is an exciting area of study for activity-aware energy models. The performance can be changed from low to high by utilizing context-aware episodic sampling [101,103].

### 6.6. Disease-Prediction-Based

The IoT helps to diagnose and treat conditions including chronic diseases, helps with geriatric care, and is used in fitness programs, by accelerating early disease detection [87]. The projected healthcare system’s future scope will advance the development of medical care that can foretell a patient’s ailment at an early stage. This disease-prediction system will shorten the time it might take to diagnose a condition and assist clinicians in providing treatment as early as possible. This will improve medical services, improve outcomes for the medical healthcare business, and lower medical costs (such as lab tests, X-rays, and some other needless medical tests). Hence, for patients’ benefit, there is also a need to develop a low-cost, independent system that tracks key indicators, transmits information to the cloud or NLP, and notifies the patient early via the appropriate APP [100,104,105].

## 7. Suggestions and Recommendations

Based on existing studies and their limitations, there is a need to enhance and integrate wearable healthcare devices to connect with other future technology trends, to solve the communication problems and drawbacks of previous studies. Researchers need to ensure that any proposed systems are user-friendly, adaptable, and secure if they want to retain satisfied customers. Disease management and healthcare can benefit from the new opportunities presented by integrating wearable sensors into healthcare systems. The IoT can provide a solution by connecting health-monitoring devices and sensors to the cloud for 24/7 monitoring. Health records are secured on the server and are available instantly.

In the future, a system could be created to diagnose patients’ conditions for chronic diseases and COVID-19; this could help doctors to make the right decision and optimize health conditions, which could improve the functionality of healthcare systems based on the IoT by combining different technological approaches.

Such integration approaches include artificial intelligence (AI), fog computing, Big Data and Nano-Things (IoNT), software-defined networks (SDNs), and the tactile Internet (TI). AI, when integrated with IoT-based healthcare-monitoring systems, can help to generate meaningful and accurate results from sensor data. The fog/edge paradigm can be used to bring computing power closer to where it is needed. Big Data computing can also be utilized in IoT healthcare-monitoring systems because Big Data can make it possible to manage extremely large amounts of data efficiently. In addition, the other most recent technologies of the future, such as the IoNT, software-defined networks (SDNs), and the tactile Internet (TI), have the potential to further enhance the functionality of IoT-based healthcare systems and expand their capabilities in the future.

## 8. Conclusions

There are endless ways in which the IoT can improve medical care. These include reduced cost, and increased efficiency, accuracy, and performance. The benefits of using the IoT have made it possible to automate healthcare systems in the best way. In this respect, this work aims to be an introductory guide for those who will work in this field in the future, providing them with a detailed reference document related to the IoT and healthcare-monitoring systems. In this work, recent research on IoT-based health-monitoring systems have been reviewed and analyzed in a systematic way. The paper provides in-depth information on their benefits and significance, and a literature review. We also discuss IoT wearable things in healthcare systems and provide a classification of health-monitoring sensors, including the challenges and open issues regarding security and privacy and Quality of Service (QoS). Suggestions for future work have also been included.

In the future, we plan to analyze and evaluate various types of disease-based classification and IoT-based healthcare-monitoring systems. We also plan, in our next phase, to stress the integration of various recent technology trends, such as SDN and AI, with IoT-based healthcare-monitoring systems.

## Data Availability

Not applicable.
